# Wavelet-Domain Information-Hiding Technology with High-Quality Audio Signals on MEMS Sensors

**DOI:** 10.3390/s22176548

**Published:** 2022-08-30

**Authors:** Ming Zhao, Shuo-Tsung Chen, Shu-Yi Tu

**Affiliations:** 1 School of Computer Science, Yangtze University, Jingzhou 434025, China; 2 Department of Applied Mathematics, Tunghai University, Taichung City 407224, Taiwan; 3 Department of Mathematics, University of Michigan, Flint, MI 48502, USA

**Keywords:** sensor, digital information, MEMS, DWT, optimization, compression

## Abstract

Due to the rapid development of sensor technology and the popularity of the Internet, not only has the amount of digital information transmission skyrocketed, but also its acquisition and dissemination has become easier. The study mainly investigates audio security issues with data compression for private data transmission on the Internet or MEMS (micro-electro-mechanical systems) audio sensor digital microphones. Imperceptibility, embedding capacity, and robustness are three main requirements for audio information-hiding techniques. To achieve the three main requirements, this study proposes a high-quality audio information-hiding technology in the wavelet domain. Due to the fact that wavelet domain provides a useful and robust platform for audio information hiding, this study applies multi-coefficients of discrete wavelet transform (DWT) to hide information. By considering a good, imperceptible concealment, we combine signal-to-noise ratio (SNR) with quantization embedding for these coefficients in a mathematical model. Moreover, amplitude-thresholding compression technology is combined in this model. Finally, the matrix-type Lagrange principle plays an essential role in solving the model so as to reduce the carrying capacity of network transmission while protecting personal copyright or private information. Based on the experimental results, we nearly maintained the original quality of the embedded audio by optimization of signal-to-noise ratio (SNR). Moreover, the proposed method has good robustness against common attacks.

## 1. Introduction

The uses of digital information transmission in Internet applications, artificial intelligence, and data sensing [1,2,3,4,5,6,7,8,9,10] are more frequent. In many cases, without permission from the legal owner, the digital information is often stolen, copied, or even turned into profit by criminal individuals. In general, an audio information-hiding technique should possess three properties: make the piece of hidden information imperceptible in the embedded audio, provide a signal-to-noise ratio (SNR) of 20 dB or more, and maintain the embedding capacity of at least 20 bps (bits per second) [11,12]. Moreover, hidden information is resistant to most attacks, which include re-sampling, MP3 compression, filtering, amplitude modification, time scaling, and so on [12,13,14,15].

Audio information-hiding techniques are classified according to their domain. These algorithms are categorized as time-domain techniques and transform-domain techniques. Discrete wavelet transform (DWT) is one practical transform domain for hiding audio information [13,14,15,16,17,18,19,20,21,22,23,24,25,26,27]. In the literature, several earlier procedures embedded watermarks into DWT low-frequency coefficients using the quantization-based technique so that they could obtain adaptive performance [15,20,24]. Chen et al. [15,24] proposed an optimization quantization approach to fixed-weighting DWT coefficients to gain high-quality modified audio and high robustness against many common attacks. Li et al. [27] proposed a new audio watermarking technique. They performed the norm ratio on fixed-scaling DWT coefficient quantization without considering the signal compression in the implementation. In addition, the quality of modified audio worsens with weighting variation, and hidden information is inadequately robust to time-scaling attacks.

This study proposes an optimization model to integrate optimization-based signal steganography [24] with threshold-based compression in the wavelet domain. Firstly, we utilized binary digits to store data and represent information. We modified the signal-to-noise ratio (SNR) and the amplitude-quantization rules in the wavelet domain as performance index and constraints. At the same time, to reduce the amount of the embedded audio data signal, we also employed threshold-based compression technology in the constrictions. Then, we obtained an optimization model that enhanced the audio quality in the information-hiding and compression processes. Secondly, the optimization model was solved by the matrix-type Lagrange principle and graphic illustration Accordingly, we performed information hiding and data compression on each audio signal for private information transmission on the Internet or MEMS (micro-electro-mechanical systems) audio sensor digital microphones. On the other end of the transmission process, the hidden information was extracted smoothly without either the original audio or the recovery of the compressed audio signal adopting a cubic spline. To demonstrate the quality of the proposed performance, we measured the appropriate threshold *ε* and embedding strength *Q* in our experiment. The proposed algorithm reduces the amount of carried network transmission but preserves the original audio signals and protects personal privacy.

The rest of this study is as follows: Section 2 presents the proposed method and introduces the embedding technique, optimization model, and compressions. The illustrations of the optimization model, presentation of the recovery method, and the extraction technique are in Section 3. Section 4 contains discissions of experimental results, and some remarks and conclusions are in Section 5.

## 2. Proposed Method

**Figure 1:**
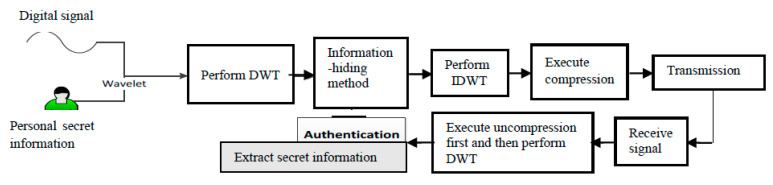
The block diagram of the proposed algorithm.

This section introduces the embedding technique, the extraction technique, and the compression of the proposed method. Figure 1 shows the block diagram of the proposed algorithm; further detailed introduction will appear in Section 2.1 and Section 2.2.

### 2.1. Embedding Technique

To embed the private information into the lowest DWT coefficients, we implemented DWT using the single prototype function ψ(x). This function is regulated by a scaling parameter and a shift parameter [28,29]. The discrete normalized scaling and wavelet basis function was defined as

(1) φi,n(t)=2i2hi(2it−n)

(2) ψi,n(t)=2i2gi(2it−n)

where i and n are the dilation and translation parameters, and $h_{i}$ and $g_{i}$ denote low-pass and high-pass filters. Orthogonal wavelet basis functions provide simple calculation of coefficient expansion and easily express audio signals $S(t)\in L^{2}(R)$ as a series expansion of orthogonal scaling functions and wavelets. Throughout this study, we used the host digital audio signal S(n), n∈N, to denote samples of the original audio signal at the *n*th sample time, and each piece of audio signal was cut into segments on which DWT was performed. As a result, the signal-to-noise ratio (SNR) 

$SNR=-10\log_{10}\left({\left\|\tilde{S}(n)-S(n)\right\|}_{2}{}^{2}/{\left\|S(n)\right\|}_{2}{}^{2}\right)$ can be rewritten as

(3) $$SNR=-10\log_{10}\left({\left\|{\tilde{X}}_{n}-X_{n}\right\|}_{2}{}^{2}/{\left\|X_{n}\right\|}_{2}{}^{2}\right)$$

where $\tilde{S}(n)$ is the modified digital audio signal and the vector form ${\tilde{X}}_{n}={\left[\begin{array}{cccc} \left|{\tilde{x}}_{1}\right| & \left|{\tilde{x}}_{2}\right| & \cdot \cdot \cdot & \left|{\tilde{x}}_{n}\right| \end{array}\right]}^{T}$ consists of the n unknown absolute values of DWT coefficients with respect to the original DWT coefficient vector $X_{n}={\left[\begin{array}{cccc} \left|x_{1}\right| & \left|x_{2}\right| & \cdot \cdot \cdot & \left|x_{n}\right| \end{array}\right]}^{T}$ in each segment. 

For convenience, the secret information is usually stored as a binary sequence. To embed the binary bit “1∈B” or “0∈B” as shown in Figure 1, we performed DWT and then determined n unknown values of DWT coefficients, ${\tilde{x}}_{1},{\tilde{x}}_{2},\cdot \cdot \cdot ,{\tilde{x}}_{n}$. Accordingly, an optimization-based model for embedding the binary bit was proposed as follows. 

We determined the vector ${\tilde{X}}_{n}$ such that the $SNR=-10\log_{10}\left({\left\|{\tilde{X}}_{n}-X_{n}\right\|}_{2}{}^{2}/{\left\|X_{n}\right\|}_{2}{}^{2}\right)$ is maximized. Due to the fact that all logarithmic functions are one-to-one, that is, for all x and y in the domain of logarithmic function, if $\log_{10}x=\log_{10}y$, then x=y. We defined a performance index of the form ${\left\|{\tilde{X}}_{n}-X_{n}\right\|}^{2}/{\left\|X_{n}\right\|}^{2}$ so that the binary sequence with binary bit “1∈B” or “0∈B” can be embedded by the proposed optimization model described below:

- If the bit “1∈B” is embedded into $X_{n}$, then $\sum_{i=1}^{n}\left|x_{i}\right|$ is quantized by
  (4a) $$minimize\frac{{\left({\tilde{X}}_{n}-X_{n}\right)}^{T}({\tilde{X}}_{n}-X_{n})}{X_{n}{}^{T}X_{n}}$$
  (4b) subjected to (a) AX˜n=⌊∑i=1n|xi|Q⌋Q+34Q 
  (b) Compression constraint;(4c)

- If the bit “0∈B” is embedded into $X_{n}$, then $\sum_{i=1}^{n}\left|x_{i}\right|$ is quantized by
  (5a) $$minimize\frac{{\left({\tilde{X}}_{n}-X_{n}\right)}^{T}({\tilde{X}}_{n}-X_{n})}{X_{n}{}^{T}X_{n}}$$
  (5b) subjected to (a) AX˜n=⌊∑i=1n|xi|Q⌋Q+14Q 
  (b) Compression constraint;(5c)

where ⌊⌋ is the floor function; Q is the quantization size or embedding strength which is adopted as the secret key K; the compression constraint is described in Equations (6)–(8) in Section 2.2. 

### 2.2. Compression Constraint

Solving the optimization models (4) and (5), the watermarked audio signal $\bar{S}$ with optimal SNR is obtained after applying the IDWT. To reduce the amount of data when transmitting on the Internet, we compressed the embedded audio signal $\bar{s}$ using the threshold compression method formulated as follows:

(6) $${\hat{s}}_{0}={\bar{s}}_{0},{\hat{s}}_{N}={\bar{s}}_{N}$$

(7) $${\hat{s}}_{i}=\{\begin{array}{lll} \phi & \mathrm{if}\left|{\bar{s}}_{i-1}-{\bar{s}}_{i+1}\right|<\varepsilon & \\ {\bar{s}}_{i} & \mathrm{otherwise} & \end{array},\;i=\left\{1,\cdots N-1\right\}$$

where ε represents the threshold.

To recover the signal ${\left\{{\bar{s}}_{i}\right\}}_{i=0}^{N}$ from the compressed signal ${\left\{{\hat{s}}_{i}\right\}}_{i=0}^{N}$, we used the cubic function, which is formulated as $f_{i}\left(t\right)=\alpha_{i}+\beta_{i}\left(t-t_{i}\right)+\gamma_{i}{\left(t-t_{i}\right)}^{2}+\eta_{i}{\left(t-t_{i}\right)}^{3}$. We found the *N* cloud-gauge line collection of functions $\left\{f_{i}\left(t\right)|i=1,\ldots ,N\right\}$ to describe the entire set of data, where $f_{i}\left(t\right)$ must satisfy

(8) $$f_{i}\left(t_{i}\right)={\hat{s}}_{i}=f_{i-1}\left(t_{i}\right),\;{f^{\prime }}_{i}\left(t_{i}\right)={f^{\prime }}_{i-1}\left(t_{i}\right),\;{f^{\prime \prime }}_{i}\left(t_{i}\right)={f^{\prime \prime }}_{i-1}\left(t_{i}\right),\;{f^{\prime \prime }}_{1}\left(t\right)={f^{\prime \prime }}_{N}\left(t\right)=0$$

To ensure the recovery quality, we adjusted the compression threshold ε while considering *Q* to better fit the optimal SNR. 

## 3. Proposed Optimization Solution in Embedding and Extraction Method

In this section, we solve the optimization problem described in models (4) and (5) in two steps. Since the optimization problems (4) and (5) are similar, we first solve (4) and then apply the optimal solution to (5) using the same method.

### 3.1. First Step in Finding the Optimal Solution

Applying Theorems A1 and A2 introduced in Appendix A, the Lagrange multiplier λ is utilized to combine (4a) and (4b) into a function *F* without any constraints,

(9) $$F({\tilde{X}}_{n},\lambda )=\frac{{\left({\tilde{X}}_{n}-X_{n}\right)}^{T}({\tilde{X}}_{n}-X_{n})}{X_{n}{}^{T}X_{n}}+\lambda \left\{A{\tilde{X}}_{n}-u_{1}\right\},\;$$

where setting ⌊∑i=1n|xi|Q⌋Q+34Q=u1. The necessary conditions for minimizing $F({\tilde{X}}_{n},\lambda )$ are

(10a) $$\frac{\partial F}{\partial {\tilde{X}}_{n}}=2({\tilde{X}}_{n}-X_{n})+A^{T}\lambda X_{n}{}^{T}X_{n}=0$$

(10b) $$\frac{\partial F}{\partial \lambda }=A{\tilde{X}}_{n}-u_{1}=0$$

Multiplying (10a) by A to observe that

(11) $$2(A{\tilde{X}}_{n}-AX_{n})+AA^{T}\lambda X_{n}{}^{T}X_{n}=0$$

Since $A{\tilde{X}}_{n}=u_{1}$, Equation (11) can be rewritten as

(12) $$u_{1}-AX_{n}+\frac{1}{2}AA^{T}\lambda =0$$

Hence, the optimal solution of λ is

(13) $$\lambda^{*}=2{\left(AA^{T}\right)}^{-1}[AX_{n}-u_{1}]$$

Moreover, by substituting (13) into (10a), the optimal DWT coefficients are

(14) $$\begin{array}{ccc} {\tilde{X}}_{n}{}^{*} & = & X_{n}-\frac{1}{2}A^{T}\lambda^{*}\\ & = & X_{n}-A^{T}{\left(AA^{T}\right)}^{-1}[AX_{n}-u_{1}] \end{array}$$

where the superscript * denotes the optimal result with respect to the corresponding variable.

### 3.2. Audio Recovery and Information Extraction

To extract the hidden confidential data, we first recover the signal ${\left\{{\bar{s}}_{i}\right\}}_{i=0}^{N}$ from the compressed signal ${\left\{{\hat{s}}_{i}\right\}}_{i=0}^{N}$ using the cubic function, which is formulated as $f_{i}\left(t\right)=\alpha_{i}+\beta_{i}\left(t-t_{i}\right)+\gamma_{i}{\left(t-t_{i}\right)}^{2}+\eta_{i}{\left(t-t_{i}\right)}^{3}$. We found that the *N* cloud gauge line collection of functions $\left\{f_{i}\left(t\right)|i=1,\ldots ,N\right\}$ to describe the entire set of data, where $f_{i}\left(t\right)$ must satisfy $f_{i}\left(t_{i}\right)={\hat{s}}_{i}=f_{i-1}\left(t_{i}\right),$${f^{\prime }}_{i}\left(t_{i}\right)={f^{\prime }}_{i-1}\left(t_{i}\right),$${f^{\prime \prime }}_{i}\left(t_{i}\right)={f^{\prime \prime }}_{i-1}\left(t_{i}\right),$${f^{\prime \prime }}_{1}\left(t\right)={f^{\prime \prime }}_{N}\left(t\right)=0$.

Next, we extract the hidden information from the DWT coefficients ${\left\{{\bar{c}}_{i}\right\}}_{i=0}^{N}$ of the recovered audio signal ${\left\{{\bar{s}}_{i}\right\}}_{i=0}^{N}$ according to the following steps:

Split the test audio into segments and perform DWT on each segment. If ${\tilde{X}}^{*}{}_{n}=\left\{\left|{\tilde{x}}^{*}{}_{1}\right|,\left|{\tilde{x}}^{*}{}_{2}\right|,\cdot \cdot \cdot ,\left|{\tilde{x}}^{*}{}_{n}\right|\right\}$ presents n consecutive DWT lowest-frequency coefficients, the binary sequence is extracted from ${\tilde{X}}^{*}{}_{n}$ by the following proposed extraction technique:

- If

(15a) ∑i=1n|x˜i*|−⌊∑i=1n|x˜i*|Q⌋Q≥Q2, 

then the extracted value is 1.

- If

(15b) ∑i=1n|x˜i*|−⌊∑i=1n|x˜i*|Q⌋Q<Q2,

then the extracted value is 0.

Finally, the hidden information is recovered from the binary sequence. In addition, to closely monitor the accuracy of the extracted private data, its ratio of bit errors (BER) is measured to check if an attack occured. The BER is usually expressed as a percentage and can be formulated as $\mathrm{BER}=\left(B_{error}/B_{total}\right)\times 100\%$, where $B_{error}$ and $B_{total}$ denote the numbers of error binary bits and total binary bits during a tested period.

### 3.3. Application Scenarios of Our Proposal

The model and techniques proposed in this study combine information hiding and data compression of audio signals. During network transmission, if the amount of data (including the hidden private information) is large and the network speed is slow, the compression ratio can be increased to save transmission time; on the contrary, if the amount of data is small or the network speed is fast, one can just perform information hiding without performing data compression to improve the accuracy of the data transmission. This is the biggest difference between the proposed method and other methods.

## 4. Experimental Results

This section presents experimental results from testing the proposed algorithm. Without loss of generality, we investigate various forms of audio signals, such as love songs, symphonies, and dance and folkloric music. Ten songs per audio were averaged to evaluate the performance of the proposed method. These mono-type signals achieved a sampling rate of 44.1 kHz, which means there were 512,000 samples in each piece of selected information. They all came with a bit depth of 16 bits and 11.6 s in length. In the embedding procedure, each audio signal with 512,000 samples was initially cut into four segments of equal length; an 8-level discrete wavelet transform was performed on each evenly cut piece. This process ensured each piece of data had the total number of lowest-frequency coefficients $512000/\left(4\cdot 2^{8}\right)=2000$. The values for *Q* are 13,000 and 26,000 for *n* = 2 and 4, respectively. To show a better comparison, we also implemented the two methods listed in references [24,27]; the experimental results are shown to compare with our algorithm.

### 4.1. Embedding Capacity and Averaged SNR

As listed in Table 1, the embedding capacities for *n* = 2 and 4 are 1000 and 500 bits, which satisfy the IFPI requirement—providing at least 20 bps (200 bits/10 s) embedding capacity. However, if the group size is greater than 16, this requirement is violated. Since we aim to present an optimization model for DWT multi-coefficients in this study, the resulting SNR of the proposed method clearly shows our SNR is much better than those SNRs using the methods in [24,27].

### 4.2. Robustness Measurement

We used five types of common attacks: re-sampling, low-pass filtering, amplitude scaling, time scaling, and MP3 compression, to evaluate how robust the proposed algorithm is. The performance quality is measured according to the averaged BER and its standard deviation (SD). A detailed discussion is illustrated below.

(1) Re-sampling: In the re-sampling process, the sampling rate of an audio signal can be increased (up-sample) or decreased (down-sample) in three stages: (i) down-sample, (ii) interpolation, and (iii) up-sample. We down-sampled the sampling rate of embedded audios from 44.1 kHz to 22.05 kHz, then up-sampled them from 22.05 kHz back to 44.1 kHz with a linear interpolation filter. A similar approach allowed the sampling rates to change from 44.1 kHz to 11.025 kHz and 8 kHz and regain the original rate of 44.1 kHz. Table 2 shows the BER of testing re-sampling on audio signals. One can see that when the re-sampling rate is 8 kHz, the proposed embedding method has lower BER than those from implementations in [24,27]. In those cases when the re-sampling rates are 22.05 kHz and 11.025 kHz, the proposed method shows comparable robustness.

(2) Low-pass filtering: Table 3 presents the BER while testing low-pass filters with cutoff frequencies of 3 kHz and 5 kHz. The BER results show that models in [24,27] have slightly higher robustness. Since both references [24,27] also adopted quantization-based embedding technique, the BER evaluation of the proposed method gives extremely similar results to theirs during the process of low-pass filtering.

(3) MPEG Audio Layer-3 (MP3) compression: Table 4 shows the BER from testing MP3 compression with different bit rates on the embedded audio data. The BER values reflect that the proposed model has similar robustness to that in references [24,27].

(4) Amplitude scaling: Since the amplitude-scaling attack usually results in saturation, in this study, we selected four distinct values for the amplitude-scaling factor: 0.5, 0.8, 1.1, and 1.2. The experimental results in Table 5 confirm that the proposed algorithm is much more robust than the methods in references [24,27].

(5) Time scaling: Table 6 lists the BER from testing time-scaling attacks with a ±2% and ±5% range. The BER results show that our method has comparable robustness to those in references [24,27].

Based on the experimental outcomes and aforementioned discussions, the proposed method generally achieves high SNR and is almost zero-error against the amplitude-scaling attacks. However, it shows slightly lower robustness against low-pass filtering attacks and poor robustness against time-scaling attacks.

### 4.3. Compression Measurement

The purpose of compression is to have maximal compression ratio (CR) under maximal SNR, where CR is defined by

$$\mathrm{CR}=\frac{\mathrm{Data}\;\mathrm{size}\;\mathrm{before}\;\mathrm{compression}}{\mathrm{Data}\;\mathrm{size}\;\mathrm{after}\;\mathrm{compression}}$$

**Figure 2:**
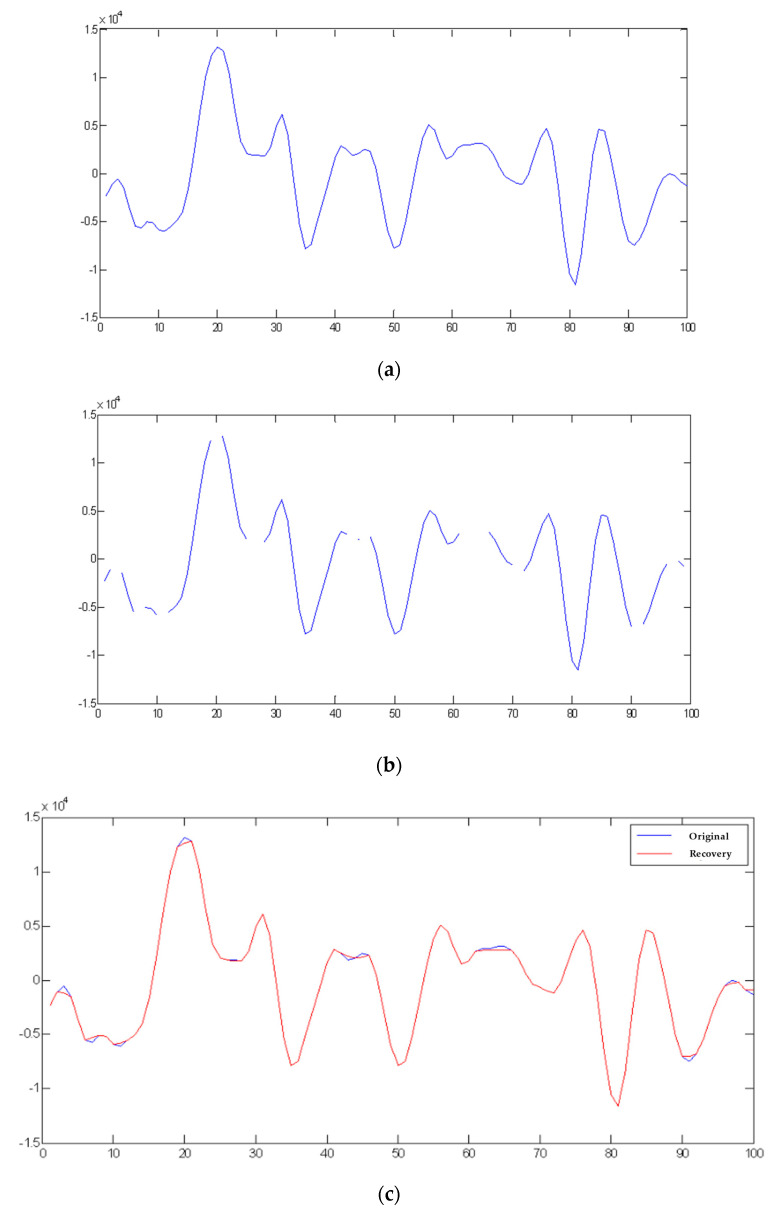

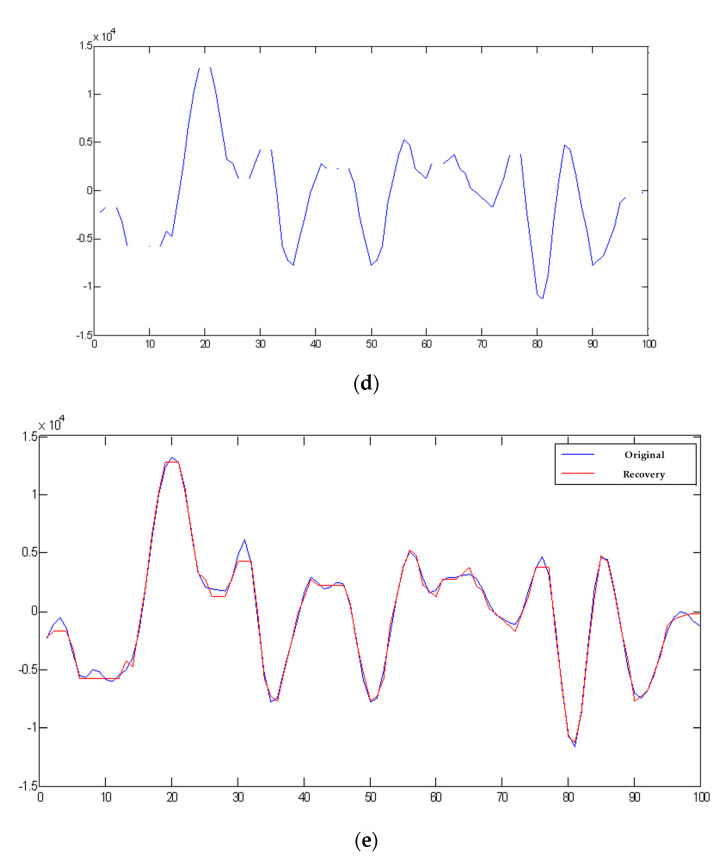
Comparison among the original audio, compressed audio, and decompressed audio in 1 and 100 audio samples with a threshold value of 500 and with/without embedding private information. (**a**) Original audio. (**b**) Compressed audio with threshold value of 500. (**c**) Recovering the compressed audio in (**b**). (**d**) Compressed audio with threshold value of 500 and embedding private information of embedding strength *Q* = 1000. (**e**) Recovering the compressed audio in (**d**).

The threshold ε and the embedding strength *Q* directly affect compression ratio (CR) and SNR, respectively. As shown in Figure 2, it can be said that the larger the threshold and the embedding strength, the larger the compression ratio CR, that is, the better the compression effect, but the worse the SNR before and after compression. 

Data in Table 7 show that the CR and SNR obtained without embedding information (denoted by N) vary under distinct threshold values. Such a result is consistent with the fact that when CR increases, SNR worsens. While signals are embedded, the result of the investigation of the relationship between the two is also in Table 7. From the experimental outcomes, we observed two noteworthy findings. First, with the same threshold value, if the embedding strength is higher, the overall audio values vary less. Though SNR seems worse, it demonstrates a more powerful embedding strength and a better compression effect. Second, in cases where threshold values change, we see that if the threshold value is greater than the embedding strength, the CR value becomes higher. That is to say, the compression effect enhancesand the relative decompression effect worsens, but the total effectiveness remains almost unchanged.

**Figure 3:**
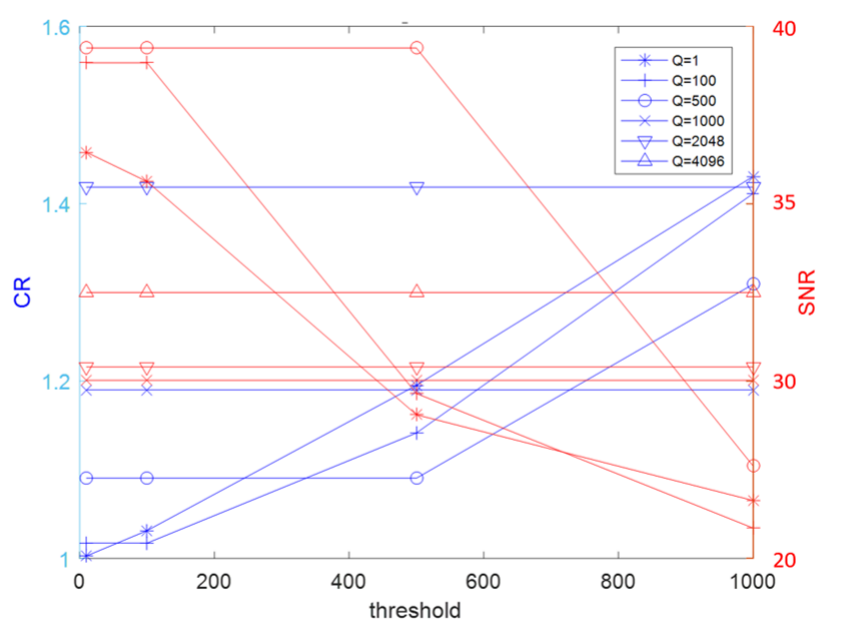
Changing the threshold ε to obtain the relationship between CR and SNR using different markers by keeping green and blue fixed to include all the *Q* values in Table 7.

Moreover, to better understand the relationship between CR and SNR, we used the graph in Figure 3 to find appropriate values of threshold *ε* and embedding strength *Q*. We changed the threshold ε to obtain the relationship between CR and SNR using different markers by keeping green and blue fixed to include all the *Q* values obtained in Table 7. By doing this, we made sure the effect between CR and SNR remained optimized.

## 5. Conclusions

This study proposes a method to seek the integration between the information-hiding process and data compression for five types of commonly seen audio signals. Under the proposed model, simulation results demonstrated that each piece of hidden audio signal attains high SNR and showed strong robustness. SNRs of most hidden audios were more than 35, and some were even higher than 40. On the other hand, most BERs were as low as 5% or less. In addition, we obtained the relationship between CR and SNR with embedded private information and observed two critical outcomes. First, with a fixed threshold value, a high embedding strength makes the differences between the overall audio values smaller. Such an algorithm shows better embedding strength and enhanced compression effects but reflects worse SNR values. Second, when playing with distinct threshold values, we found that if the threshold value *ε* is set higher than the embedding strength *Q*, the CR value drops. That means the compression effect becomes better and the relative decompression effect worsens, but the total effectiveness remains almost unchanged.

## Figures and Tables

**Table 1:** Embedding capacity and SNR.

	Number of Consecutive Coefficients in DWT Level 8	Embedding Capacity (bits/11.6 s)	Averaged SNR (dB)			
			Dance	Love Song	Folklore	Symphony
Reference [24]	*n* = 2	1000	35.8	33.4	27.9	26.3
	*n* = 4	500	37.7	33.5	28.6	26.2
Reference [27]	*n* = 2	1000	24.3	25.4	23.2	22.3
	*n* = 4	500	24.1	26.0	23.6	22.9
Proposed Method	*n* = 2	1000	38.3	35.6	28.7	27.5
	*n* = 4	500	37.1	41.3	34.5	33.2

**Table 2:** BER of Testing Re-sampling.

Audio Type			Dance			Folklore			Love Song			Symphony		
Re-Sampling Rate (kHz)			22.05	11.025	8	22.05	11.025	8	22.05	11.025	8	22.05	11.025	8
Reference [24]	*n* = 2	mean	8.32	13.31	14.01	0.74	4.22	4.36	5.74	3.08	2.39	0.78	4.52	4.76
		SD	0.40	0.43	0.41	0.23	0.28	0.26	0.16	0.15	0.16	0.16	0.26	0.28
	*n* = 4	mean	2.36	8.01	8.01	0.20	1.24	1.26	0.72	1.05	1.06	0.32	1.29	1.29
		SD	0.25	0.38	0.36	0.18	0.21	0.19	0.10	0.12	0.12	0.13	0.21	0.21
Reference [27]		mean	9.14	15.26	15.31	0.74	4.22	4.36	5.74	3.08	2.39	0.78	4.52	4.76
	*n* = 2	SD	0.41	0.43	0.42	0.19	0.24	0.27	0.17	0.14	0.13	0.14	0.27	0.25
	*n* = 4	mean	2.17	8.03	8.04	0.23	1.21	1.31	0.62	1.21	1.02	0.35	1.27	1.28
		SD	0.21	0.39	0.37	0.15	0.20	0.19	0.11	0.12	0.11	0.13	0.22	0.21
Proposed method	*n* = 2	mean	8.25	14.42	0.87	0.82	4.65	4.35	4.87	3.29	0.68	0.66	1.28	1.28
		SD	0.39	0.4	0.16	0.18	0.22	0.21	0.15	0.16	0.09	0.14	0.21	0.21
	*n* = 4	mean	2.1	8.16	0.26	0.23	1.42	1.25	1.34	1.45	0.57	0.53	1.24	1.23
		SD	0.23	0.38	0.14	0.16	0.19	0.15	0.13	0.11	0.08	0.12	0.20	0.19

**Table 3:** BER of Testing Low-pass Filtering.

Audio Type			Love Song		Symphony		Dance		Folklore	
Cutoff Frequency			3 kHz	5 kHz	3 kHz	5 kHz	3 kHz	5 kHz	3 kHz	5 kHz
Reference [24]	*n* = 2	mean	24.18	25.82	27.58	8.68	33.62	21.52	33.62	15.72
		SD	0.28	0.22	0.29	0.20	0.35	0.29	0.34	0.21
	*n* = 4	mean	23.82	23.48	27.55	8.41	33.25	21.28	33.02	13.84
		SD	0.27	0.19	0.28	0.20	0.36	0.27	0.34	0.19
Reference [27]	*n* = 2	mean	26.18	25.82	27.53	8.68	33.62	21.52	33.62	15.72
		SD	0.29	0.21	0.28	0.19	0.35	0.27	0.33	0.19
	*n* = 4	mean	25.82	24.81	27.54	8.41	33.02	20.87	33.02	11.84
		SD	0.29	0.21	0.25	0.21	0.34	0.28	0.33	0.17
Proposed method	*n* = 2	mean	22.84	23.63	27.85	8.38	32.28	20.03	31.82	13.32
		SD	0.27	0.19	0.27	0.18	0.35	0.26	0.29	0.15
	*n* = 4	mean	21.42	23.63	27.54	8.25	30.39	20.02	32.50	13.15
		SD	0.25	0.18	0.24	0.19	0.33	0.25	0.30	0.16

**Table 4:** BER of Testing MP3 compression.

Audio Type			Love Song				Symphony				Dance				Folklore			
Bit Rate (kbps)			128	112	96	80	128	112	96	80	128	112	96	80	128	112	96	80
Reference [24]	*n* = 2	mean	0.16	1.38	2.09	2.72	0.35	1.45	2.44	3.17	0.74	2.11	2.12	3.02	0.36	1.48	2.42	3.12
		SD	0.11	0.11	0.13	0.15	0.12	0.13	0.14	0.17	0.15	0.18	0.18	0.21	0.12	0.15	0.14	0.16
	*n* = 4	mean	0.09	0.11	1.41	2.53	0.14	0.15	2.29	3.84	0.11	0.15	1.02	3.0	0.15	0.15	2.40	3.93
		SD	0.10	0.09	0.12	0.15	0.10	0.10	0.13	0.16	0.12	0.13	0.17	0.23	0.11	0.10	0.13	0.17
Reference [27]	*n* = 2	mean	0.15	1.32	2.13	2.73	0.27	1.45	2.44	3.17	0.75	2.13	2.16	3.02	0.36	1.46	2.43	3.14
		SD	0.11	0.13	0.13	0.14	0.13	0.14	0.15	0.15	0.14	0.18	0.19	0.20	0.13	0.13	0.14	0.15
	*n* = 4	mean	0.09	0.11	1.42	2.53	0.14	0.17	2.32	3.74	0.11	0.16	1.02	3.0	0.15	0.13	2.40	3.02
		SD	0.10	0.10	0.11	0.15	0.12	0.09	0.13	0.15	0.13	0.15	0.15	0.20	0.12	0.08	0.13	0.15
Proposed method	*n* = 2	mean	0.75	2.67	2.91	3.31	0.18	0.15	2.29	3.92	0.83	2.46	2.54	2.62	0.45	2.13	2.64	3.25
		SD	0.13	0.14	0.14	0.15	0.14	0.08	0.12	0.15	0.16	0.18	0.19	0.19	0.14	0.12	0.12	0.16
	*n* = 4	mean	0.69	2.23	2.24	2.28	0.17	0.12	1.93	2.09	0.15	0.13	2.48	2.49	0.39	1.94	1.95	1.94
		SD	0.12	0.14	0.13	0.13	0.13	0.06	0.09	0.12	0.15	0.15	0.16	0.16	0.13	0.13	0.09	0.10

**Table 5:** BER of Testing Amplitude Scaling.

Audio Type		Love Song				Symphony				Dance				Folklore			
Amplitude Modification Factor		0.5	0.8	1.1	1.2	0.5	0.8	1.1	1.2	0.5	0.8	1.1	1.2	0.5	0.8	1.1	1.2
Reference [24]	*n* = 2	47.25	45.55	41.40	43.85	48.00	38.72	23.63	24.54	43.12	41.40	40.15	40.84	45.90	43.52	42.54	42.86
	*n* = 4	43.82	40.63	40.84	41.25	45.22	32.04	23.15	23.56	42.33	41.02	39.56	40.16	42.52	41.86	41.35	41.24
Reference [27]	*n* = 2	40.02	32.15	31.18	33.65	38.06	31.22	28.13	28.55	38.92	31.41	32.10	34.24	39.82	33.12	32.74	32.62
	*n* = 4	38.22	30.63	30.84	31.25	35.22	32.04	23.15	23.56	40.02	31.11	30.51	30.46	32.42	26.81	24.75	24.26
Proposed method	*n* = 2	2.03	1.15	1.08	1.13	1.65	0.97	1.43	1.45	2.85	1.76	1.85	2.06	1.67	1.31	0.93	1.32
	*n* = 4	0.97	0.86	0.84	0.92	1.14	0.88	0.92	0.98	2.04	1.56	0.98	1.93	1.05	0.86	0.83	0.85

**Table 6:** BER of Testing Time Scaling.

Audio Type			Love Song				Symphony				Dance				Folklore			
Time-Scaling (%)			−5	−2	2	5	−5	−2	2	5	−5	−2	2	5	−5	−2	2	5
Reference [24]	*n* = 2	mean	47.11	42.91	43.67	46.32	42.74	37.82	46.42	46.19	45.18	40.21	46.58	47.98	43.18	39.12	46.35	47.43
		SD	0.10	0.09	0.08	0.09	0.11	0.11	0.09	0.10	0.12	0.12	0.11	0.13	0.12	0.12	0.10	0.12
	*n* = 4	mean	47.04	40.23	45.11	46.58	43.11	36.64	46.24	46.86	44.37	39.91	44.92	47.98	43.03	38.62	46.53	47.54
		SD	0.08	0.07	0.07	0.08	0.09	0.10	0.09	0.10	0.12	0.11	0.11	0.12	0.13	0.12	0.09	0.10
Reference [27]	*n* = 2	mean	48.24	45.03	41.13	42.62	42.24	40.73	43.62	45.21	46.29	42.07	44.98	45.18	44.15	40.22	45.39	47.37
		SD	0.09	0.08	0.07	0.08	0.09	0.10	0.08	0.09	0.13	0.13	0.12	0.14	0.13	0.12	0.11	0.11
	*n* = 4	mean	46.12	41.25	44.01	44.52	42.01	38.34	45.27	45.89	45.27	40.91	45.02	46.13	42.53	39.24	45.63	45.58
		SD	0.08	0.08	0.08	0.07	0.10	0.09	0.09	0.09	0.12	0.13	0.11	0.14	0.13	0.11	0.10	0.09
Proposed method	*n* = 2	mean	47.23	42.05	43.53	45.15	42.32	37.64	45.18	46.21	45.35	40.42	46.24	46.47	43.18	38.93	46.41	47.13
		SD	0.07	0.07	0.06	0.07	0.08	0.09	0.09	0.10	0.11	0.10	0.10	0.13	0.12	0.11	0.08	0.09
	*n* = 4	mean	46.43	40.08	44.37	46.54	43.06	36.83	46.32	46.25	44.14	39.65	44.78	47.95	42.25	38.26	46.42	46.37
		SD	0.07	0.05	0.05	0.06	0.08	0.09	0.07	0.08	0.11	0.11	0.10	0.12	0.10	0.10	0.08	0.08

**Table 7:** Relationship between CR and SNR with and without embedding private information (*N*).

Threshold ε	*Q*	CR	SNR before Decompression
0.1	1	1.0016	36.2503
	100	1.0173	38.9726
	500	1.0905	31.7549
	1000	1.1900	28.0046
	2048	1.4187	23.0792
	4096	1.8970	17.4999
10	1	1.0028	35.2693
	100	1.0173	38.9726
	500	1.0905	31.7549
	1000	1.1900	28.0046
	2048	1.4187	23.0792
	4096	1.8970	17.4999
100	1	1.0314	34.1682
	100	1.0173	38.9726
	500	1.0905	31.7549
	1000	1.1900	28.0046
	2048	1.4187	23.0792
	4096	1.8970	17.4999
500	1	1.1953	25.4036
	100	1.1415	29.6514
	500	1.0905	31.7549
	1000	1.1900	28.0046
	2048	1.4187	23.0792
	4096	1.8970	17.4999
1000	1	1.4308	22.1784
	100	1.4115	25.8566
	500	1.3092	27.0426
	1000	1.1900	28.0046
	2048	1.4187	23.0792
	4096	1.8970	17.4999

## Data Availability

Not applicable.

## Appendix A

**Theorem** **A1.** *Let A be a matrix of size n × n. If *$\bar{X}$*and X are n × 1 column vectors, then the following statement holds* [30,31]:

$$\frac{\partial A\overset{¯}{X}}{\partial \overset{¯}{X}}=A,$$

$$\frac{\partial {\left(\overset{¯}{X}-X\right)}^{T}\left(\overset{¯}{X}-X\right)}{\partial \overset{¯}{X}}=2\left(\overset{¯}{X}-X\right).$$

**Theorem** **A2.** *Suppose that g is a continuously differentiable function of X on a subset of the domain of a function f. If X*_0_*minimizes (or maximizes) f(X) subjected to the constraint g*(*X*) = 0, *then* ∇*f*(*X*_0_) *and* ∇*g*(*X*_0_) *are parallel and one is a constant multiple of another. That is, if* ∇*g*(*X*_0_) ≠ 0, *then there exists a non-zero scalar λ such that*

$$\nabla f\left(X_{0}\right)=\lambda \nabla g\left(X_{0}\right).$$

Based on Theorem A2, if an augmented function is defined as

H(X,λ)=f(X)+λg(X),

then an optimal solution of the optimization problem is to compute the extreme of the unconstraint function *H*(*X*, *λ*). The necessary conditions to ensure the existence of the extreme of *H* are [30,31]

$$\frac{\partial H}{\partial \lambda }=0,\frac{\partial H}{\partial X}=0$$
